# Assessment of mangroves from Goa, west coast India using DNA barcode

**DOI:** 10.1186/s40064-016-3191-4

**Published:** 2016-09-13

**Authors:** Ankush Ashok Saddhe, Rahul Arvind Jamdade, Kundan Kumar

**Affiliations:** 1 Department of Biological Sciences, Birla Institute of Technology and Science Pilani, K. K. Birla Goa Campus, Sancoale, Goa 403726 India; 2 Department of Zoology, Yashwantrao Chavan Institute of Science, Satara, Maharashtra 415001 India

**Keywords:** Mangrove, Goa, DNA barcode, *rbcL*, *matK*

## Abstract

Mangroves are salt-tolerant forest ecosystems of tropical and subtropical intertidal regions. They are among most productive, diverse, biologically important ecosystem and inclined toward threatened system. Identification of mangrove species is of critical importance in conserving and utilizing biodiversity, which apparently hindered by a lack of taxonomic expertise. In recent years, DNA barcoding using plastid markers *rbcL* and *matK* has been suggested as an effective method to enrich traditional taxonomic expertise for rapid species identification and biodiversity inventories. In the present study, we performed assessment of available 14 mangrove species of Goa, west coast India based on core DNA barcode markers*, rbcL* and *matK*. PCR amplification success rate, intra- and inter-specific genetic distance variation and the correct identification percentage were taken into account to assess candidate barcode regions. PCR and sequence success rate were high in *rbcL* (97.7 %) and *matK* (95.5 %) region. The two candidate chloroplast barcoding regions (*rbcL, matK*) yielded barcode gaps. Our results clearly demonstrated that *matK* locus assigned highest correct identification rates (72.09 %) based on TaxonDNA Best Match criteria. The concatenated *rbcL* + *matK* loci were able to adequately discriminate all mangrove genera and species to some extent except those in *Rhizophora, Sonneratia* and *Avicennia*. Our study provides the first endorsement of the species resolution among mangroves using plastid genes with few exceptions. Our future work will be focused on evaluation of other barcode markers to delineate complete resolution of mangrove species and identification of putative hybrids.

**Electronic supplementary material:**

The online version of this article (doi:10.1186/s40064-016-3191-4) contains supplementary material, which is available to authorized users.

## Background

Mangroves are unique ecosystem exist along the sheltered inter-tidal coastline, in the margin between the land and sea in tropical and subtropical areas. This ecosystem endowed with productive wetland having flora and fauna adapted to local environment such as fluctuated water level, salinity and anoxic condition (Tomlinson 1986; Hutchings and Saenger 1987). They are most productive and biologically important ecosystems of the world which provide goods and services to human society in coastal and marine systems (FAO 2007). They have unique features such as aerial breathing roots, extensive supporting roots, buttresses, salt-excreting leaves and viviparous propagules (Duke 1992; Shi et al. 2006). The term ‘mangroves’ are referred to either individual plant or intertidal ecosystem or both, as ‘Mangrove plants’ and ‘Mangrove ecosystem’ (MacNae 1968). However, in this context we used mangrove term as a mangrove plants. Anthropogenic activity and climate are responsible for destruction of coastal mangroves vegetation. Globally among 11 of the 70 mangrove species were listed threatened species by International Union for Conservation of Nature (IUCN) (Polidoro et al. 2010).

Mangrove species diversity and distribution reported existence of 34 major and 20 minor mangrove species belonging to 20 genera and 11 families across the world (Tomlinson 1986). Ricklefs and Latham (1993) reported the existence of 19 genera with 54 mangrove species including few hybrids. According to world atlas of mangroves database, 73 mangrove species along with few recognized hybrids are distributed in 123 countries with territorial coverage of 150,000 km^2^ area globally (Spalding et al. 2010). Indian mangrove vegetation represents fourth largest in the world, distributed along the coastline and occupies 8 % of the total world mangrove covering 6749 km^2^ areas (Naskar and Mandal 1999). The entire mangrove habitats in India are situated in three zones: east coast (4700 km^2^), west coast (850 km^2^) and Andaman & Nicobar Islands (1190 km^2^). East coast zone ranges from Sundarban forest of West Bengal to Cauvery estuary of Tamil Nadu and comprises 70 % mangrove (Untawale and Jagtap 1992; Jagtap et al. 1993; Sanyal et al. 1998). West coast region stretches from Bhavnagar estuary of Gujarat to Cochin estuary of Kerala and constitute 15 % mangrove (Mandal and Naskar 2008). Mangrove flora of India constitutes about 60 species belonging to 41 genera and 29 families (Untawale 1985). Along the west coast of India, 34 species of mangroves belonging to 25 genera and 21 families have been reported. There are about 11, 20, 14 and 10 species of mangroves reported along the coast of Gujarat, Maharashtra, Goa and Karnataka respectively in western India. Goa state is located in western coast of India and mangrove vegetation in Goa occupies 500 ha of area (Government of India, 1997). The Cumbarjua canal (15 km) links the two river channels of Mandovi and Zuari, forming an estuarine complex which supports a substantial mangrove extent. D’Souza and Rodrigues (2013) reported the presence of 17 mangrove species in Goa that include 14 true and 3 associated mangrove species.

DNA barcoding is currently used effective tool that enables rapid and accurate identification of plant (Li et al. 2015). The Consortium for the Barcode of Life (CBOL) recommended *rbcL* + *matK* as the core barcode. However, these core barcode further combined with the *psb*A-*trn*H intergenic non-coding spacer region which improved discrimination power of core barcode. The non-coding intergenic region *psb*A-*trn*H exhibits high rates of insertion/deletion and sequence divergence (Kress and Erickson 2007). These features make *trnH*-*psbA* highly suitable candidate plant barcode for species resolution. Later on, the nuclear ribosomal internal transcribed spacer (ITS) region considered as supplementary barcode, though China Plant Barcode of Life claimed ITS region had higher discriminatory power than plastid core barcodes (CBOL Plant Working Group 2009; Hollingsworth et al. 2011; China Plant BOL Group 2011). Hollingsworth et al. (2011) observed ITS region has some limitations which prevent it from being a core barcode such as incomplete concerted evolution, fungal contamination and difficulties of amplification and sequencing. Plastid gene large subunit of the ribulose-bisphosphate carboxylase gene (*rbcL*) is of 1350 bp in length and choice for DNA barcoding (Chase 1993).The maturase gene *matK* is about 1500 bp long and located within the *trnK* gene encoding the tRNALys (UUU). Substitution rate of the *matK* gene is highest among the plastid genes (Hilu et al. 2003). Plastid gene *matK* can discriminate more than 90 % of species in the Orchidaceae but less than 49 % in the nutmeg family (Kress and Erickson 2007; Newmaster et al. 2008). In another case, identification of 92 species from 32 genera using the *matK* barcode could achieve a success rate of 56 % (Fazekas et al. 2008). However, a recent study of the flora of Canada revealed 93 % success in species identification with *rbcL* and *matK*, while the addition of the trnH-psbA intergenic spacer achieved discrimination up to 95 % (Burgess et al. 2011). Gonzalez et al. (2009) reported that species discrimination was lower (<50 %) for *rbcL* + *matK* combination in the study of tropical tree species in French Guiana. Lower discrimination were reported in closest and complex taxa of *Lysimachia*, *Ficus*, *Holcoglossum* and *Curcuma* using *rbcL* and *matK* (Xiang et al. 2011; Zhang et al. 2012; Li et al. 2012; Chen et al. 2015). The lowest discriminatory power was observed in closely related groups of *Lysimachia* with *rbcL* (26.5–38.1 %), followed by *matK* (55.9–60.8 %) and combinations of core barcodes (*rbcL* + *matK*) had discrimination of 47.1–60.8 % (Zhang et al. 2012).

Delineating mangrove species from putative hybrids using morphological characters are always questionable. Putative hybrids were reported within the major genera of *Rhizophora*, *Sonneratia* and *Lumnitzera* and recently in *Bruguiera* (Tomlinson 1986; Duke and Ge 2011). In the present study, we assessed mangrove species using plastid coding loci viz. *rbcL* and *matK*. Mangroves from Goa are rich in diversity and accounted 14 species belonging to four order and five families. This is our first step towards DNA barcoding of mangroves based on plastid genes. Our study might be helpful in identification as well as developing various strategies towards mangrove conservation.

## Methods

### Sample collection

In the present study, leaf samples of 14 mangrove species were collected from Goa, located on the west coast of India with geographical latitude of 15.5256°N and longitude of 73.8753°E. Mangrove species identification was performed based on morphological characteristics using a comparative guide to the Asian mangroves and mangroves of Goa (Yong and Sheue 2014; Dhargalkar et al. 2014; Setyawan et al. 2014). Herbarium of these specimens was deposited at Botanical Survey of India, western regional centre, Pune, India. The morphology based identification keys used to authenticate the taxon identities of 14 mangroves species from Goa were listed in supplementary information (Additional file 1: Table S1). The well identified voucher specimens along with their taxonomic information and collection details are listed (Table 1) with their photographs in supplementary information (Additional file 1: Fig. S1). The sequences obtained using barcode markers: *rbcL* and *matK* were submitted to the NCBI GenBank (Accession numbers indicated in Table 1), and publicly accessible through the dataset of project DNA Barcoding of Indian Mangroves (Project code: IMDB) in Barcode of Life Data systems (BOLD) (doi:10.5883/DS-IMDBNG) (Ratnasingham and Hebert 2007).

**Table 1:** Details of the mangrove species used in the present study with family, status, life form, voucher number and GenBank accession numbers obtained after sequence submission

S. No.	Specimen	Family	Status	Life form	Herbarium Voucher No.	Accession No. *rbcL*	Accession No. *matK*
1	*Avicennia officinalis*	Acanthaceae	TM	Tree	AAS-100-02	KP697351, KP697352, KU748517	KP725238, KP725239
2	*Avicennia marina*	Acanthaceae	TM	Tree	AAS-110-12	KP697349, KP697350, KM255068	KP725236, KM255083, KP725237
3	*Avicennia alba*	Acanthaceae	TM	Tree	AAS-120-22	KM255067, KM255069, KP697348	KM255082, KM255084, KP725235
4	*Bruguiera cylindrica*	Rhizophoraceae	TM	Tree	AAS-130-32	KP697354, KM255070, KP697353	KP725241, KM255085, KP725240
5	*Bruguiera gymnorrhiza*	Rhizophoraceae	TM	Tree	AAS-140-42	KM255071,KP697355,KP697356	KM255086,KP725242,KP725243
6	*Rhizophora mucronata*	Rhizophoraceae	TM	Tree	AAS-150-52	KM255077, KU748519	KM255092, KU748522, KU748523
7	*Rhizophora apiculata*	Rhizophoraceae	TM	Tree	AAS-160-62	KP697362, KP697363, KM255076	KP725249, KP725250, KM255091
8	*Aegiceras corniculatum*	Primulaceae	MM^T^	Tree/Shrub	AAS-170-72	KM255066, KP697344, KP697345, KM255075, KP697346, KP697347	KM255081, KP725231, KP725232, KM255090, KP725233, KP725234
9	*Excoecaria agallocha*	Euphorbiaceae	TM	Tree	AAS-180-82	KM255073, KP697360, KP697359	KM255088, KP725247, KP725246
10	*Kandelia candel*	Rhizophoraceae	TM	Tree	AAS-190-92	KP697361, KM255074, KU748518	KP725248, KM255089, KU748521
11	*Ceriops tagal*	Rhizophoraceae	TM	Tree	AAS-200-02	KM255072, KP697358, KP697357	KM255087, KP725244, KP725245
12	*Sonneratia alba*	Lythraceae	TM	Tree	AAS-210-12	KM255078, KP697364, KU748520	KM255093, KP725251
13	*Sonneratia caseolaris*	Lythraceae	TM	Tree	AAS-220-22	KP697365, KP697366, KM255079	KP725252, KP725253, KM255094
14	*Acanthus ilicifolius*	Acanthaceae	TM	Shrub	AAS-230-32	KM255065, KP697342, KP697343	KM255080, KP725229, KP725230

*TM* True Mangroves, *MM* Minor Mangroves, *T* Tomlinson (1986)

### DNA extraction

High content of mucilage, latex, phenolics, secondary metabolites and polysaccharides in these plants make it a difficult system for protein and nucleic acid isolation from mangrove plants. Cetyl-trimethyl ammonium bromide (CTAB) protocol for DNA extraction from mangroves (Parani et al. 1997a) was modified. Leaf tissue was pulverized in liquid nitrogen and pulverized leaf sample (0.2 g) were mixed with CTAB buffer (20 mM EDTA; 1.4 M NaCl; 2 % PVP-30; 1 % β-mercaptoethanol; 10 % SDS and 10 mg/ml proteinase K). The suspension was incubated at 60 °C for 60 min with gentle mixing and centrifuged at 14,000 rpm for 10 min at room temperature with equal volume of chloroform: isoamyl alcohol (24:1). The aqueous phase was transferred to a new tube and DNA was precipitated with 0.6 volume of cold isopropanol (−20 °C) and chilled 7.5 M ammonium acetate followed by storing at −20 °C for 1 h. The precipitated DNA was centrifuged at 14,000 rpm for 10 min at 4 °C followed by washing with 70 % ethanol. DNA was finally dissolved in TE buffer (10 mM Tris–HCl, 1 mM Na_2_EDTA, pH 8.0) and its quantity and quality was confirmed by agarose gel electrophoresis and nanodrop (Thermo Scientific, USA).

### PCR and sequencing

Amplification of plastid genes (*rbcL* and *matK*) was carried out in 50-μl reaction mixture containing 10–20 ng of template DNA, 200 μM of dNTPs, 0.1 μM of each primers and 1 unit of Taq DNA polymerase (Thermo Scientific, USA). The reaction mixture was amplified in Bio-Rad (T100 model) thermal cycler with temperature profile for *rbcL* (94 °C for 4 min; 35 cycles of 94 °C for 30 s, 55 °C for 30 s, 72 °C for 1 min; repeated for 35 cycles, final extension 72 °C for 10 min) and for *matK* (94 °C for 1 min; 35 cycles of 94 °C for 30 s, 50 °C for 40 s, 72 °C for 40 s; repeated for 37 cycles, final extension 72 °C for 5 min). The amplified products were separated by agarose gel (1.2 %) electrophoresis and stained with ethidium bromide (Sambrook et al. 1989). Two pair of universal primers *rbcL* (*rbcL*a_F and *rbcL*a_R) and *matK*_390f and *matK*_1326r were used for the amplification purpose (Kress and Erickson 2007; Vinitha et al. 2014; Chen et al. 2015). To amplify *R. apiculata matK* locus, we designed *matK*_RA reverse primer as follows: 5′-AAAGTTCGTTTGTGCCAATGA-3′. PCR products were purified according to manufacturer’s instruction (Chromous Biotech) and further sequencing reactions were carried out using the Big Dye Terminator v3.1 Cycle Sequencing Kit (Applied Biosystems) and analyzed on ABI 3500xL Genetic Analyzer (Applied Biosystems).

### Data analysis

Sequence alignment and assembly was achieved in Codon code Aligner v.3.0.1 (Codon Code Corporation) and MEGA 6 (Tamura et al. 2013). The NCBI BLAST was performed to confirm identity of specimens (Altschul et al. 1990). All known mangroves sequences were searched with our sequenced samples using ‘BLASTn’ tool against NCBI database and highest-scoring hit from each query is taken as the mangrove identification. Intraspecific, interspecific and barcode gap analysis was performed at Barcode of Life Data systems web portal. Further, *rbcL* and *matK* sequences were concatenated using DNASP v5.10 and analyzed in MEGA 6 for their resolution inference (Rozas, 2009). The effectiveness of the analysed barcodes in *rbcL*, *matK* and *rbcL* + *matK* was evaluated using TaxonDNA v1.6.2, Species Identifier 1.8 (Meier et al. 2006) and BLASTClust (http://toolkit.tuebingen.mpg.de/blastclust). Neighbor-joining (NJ) trees were constructed using MEGA 6.0 and K2P genetic distance model, and node support was assessed based on 1000 bootstrap replicates. Species with multiple individuals forming a monophyletic clade in phylogenetic trees with a bootstrap value above 60 % were considered as successful identification.

## Results

### DNA barcode and sequence analysis

Mangroves belonging to 14 species, 9 genera and 5 families were collected. We acquired high quality DNA barcodes for 45 specimens belonging to 14 species, which were sequenced for *rbcL* and *matK*. The sequencing result of *rbcL* produced an average of 510 bp without any insertion, deletion and stop codon, whereas *matK* sequencing produced 712 bp with few insertion and deletions in the form of gaps without stop codon. Overall GC content observed in *rbcL* was 43.29 % (SE = 0.09), while in *matK* it was 33.18 % (SE = 0.18). The mean GC content of codon at positions 1-3 in *rbcL* was 54.66 % (SE = 0.1), 45.77 % (SE = 0.09) and 29.44 % (SE = 0.21), and in *matK,* it was 33.15 % (SE = 0.18), 30.92 % (SE = 0.36), 29.91 % (SE = 0.25) respectively. The specimen data, collection site details and sequences were submitted to BOLD database in form of project IMDB (doi:10.5883/DS-IMDBNG) (For details, Table 1). The specimens were verified from sequenced data by performing NCBI BLAST. This is performed for preliminary verification for all mangroves at species level but downside in our case study is limited reference data for comparison. The *rbcL* and *matK* correctly identified genera up to 100 %, while species identification with *rbcL* and *matK* leads to 64 and 85 % identification respectively.

### Intraspecific and interspecific relationship

Barcoding of mangrove exhibited absolute average interspecific differentiation of 0.35 % and 0.9 % in *rbcL* and *matK* respectively, while for species average intraspecific variability was 0.24 % in *rbcL* and 0.20 % in *matK* (Table 2) with low species resolution in few taxa. The intraspecific and interspecific analysis for *rbcL* revealed largest average pairwise distance of 0.68, while in *matK* it was 2.05 and 2.32 respectively. The highest range of congeneric differentiation in *Bruguiera* and *Avicennia* were observed in *rbcL* from 0 to 0.68, whereas for *matK,* it ranged from 1.29 to 2.31 in *Avicennia,* further suggesting significant genetic divergence within *Avicennia.*

**Table 2:** Genetic divergence of mangrove species based on Kimura 2 Parameter within species, genus and family levels

	No. of sequences	Taxa	Comparisons	Min Dist (%)	Mean Dist (%)	Max Dist (%)	SE Dist (%)
For *rbcL*							
Within species	44	14	53	0	0.24	0.68	0
Within genus	26	4	50	0	0.35	0.68	0
Within family	29	2	132	1.71	2.63	4.01	0
For *matK*							
Within species	43	14	50	0	0.2	1.32	0.01
Within genus	25	4	45	0	0.9	2.32	0.02
Within family	29	2	141	2.11	5.82	13.37	0.02

*Min Dist* Minimum distance, *Max Dist* Maximum distance, *SE Dist* Standard error distance

### Barcode gap analysis

The barcode gap analysis revealed highest intraspecific distance (>2 %) in 9 specimens of *rbcL* and 6 specimens of *matK*, while low intraspecific distance (<2 %) in 11 specimens of *rbcL* and 9 specimens of *matK*. Here, low intraspecific distance (<2 %) suggests low species resolution, thus leading to species overlap.

**Fig. 1:**
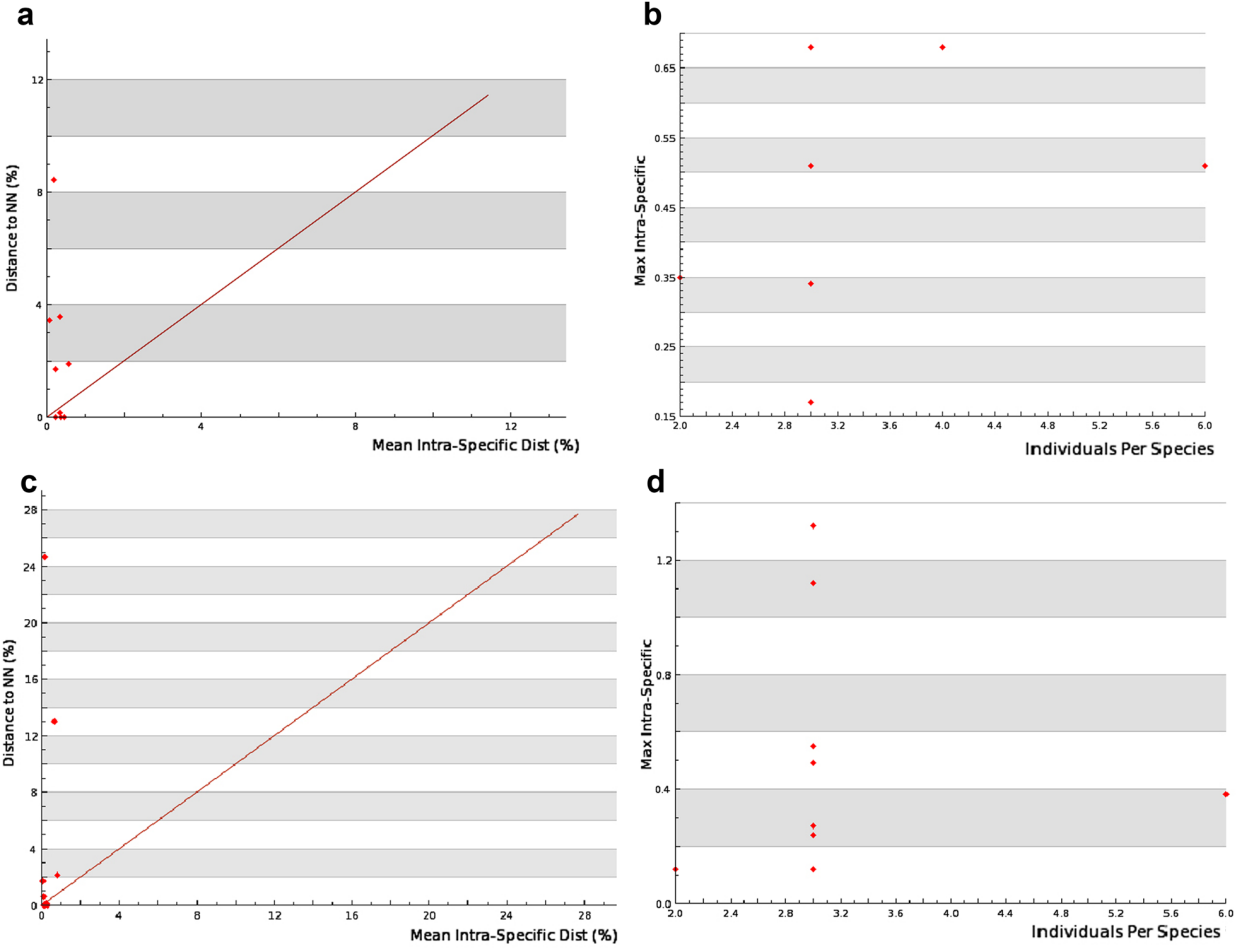
Scatterplots confirming the existence and magnitude of the barcode gap. **a** For *rbcL* mean intra-specific versus Nearest Neighbor (NN). **b** For *rbcL* individuals per species vs. max intra-specific. **c** For *matK* mean intra-specific versus Nearest Neighbour. **d** For *matK* individuals per species versus max intra-specific

With *rbcL* the largest nearest neighboring distance of 8.43 was observed in *Avicennia alba* with mean intraspecific distance of 0.11 (Fig. 1a). The maximum intraspecific distance of 0.68 was observed within three individuals of *Kandelia candel, Bruguiera gymnorrhiza, A. officinalis* and *Sonneratia caseolaris* (Fig. 1b). With *matK*, maximum intraspecific distance of 2.05 was observed in *Excoecaria agallocha* with three individuals per species (Fig. 1d), while largest distance to the nearest neighbor of 24.65 was observed in *A. officinalis* with mean intraspecific distance of 0.12 (Fig. 1c). Overall average nearest neighboring divergence observed among mangroves using *rbcL* was 1.39 % (S.E = 0.17) and *matK* was 4.07 % (S.E = 0.5) (Fig. 1a).

### Species identification and assignment

**Fig. 2:**
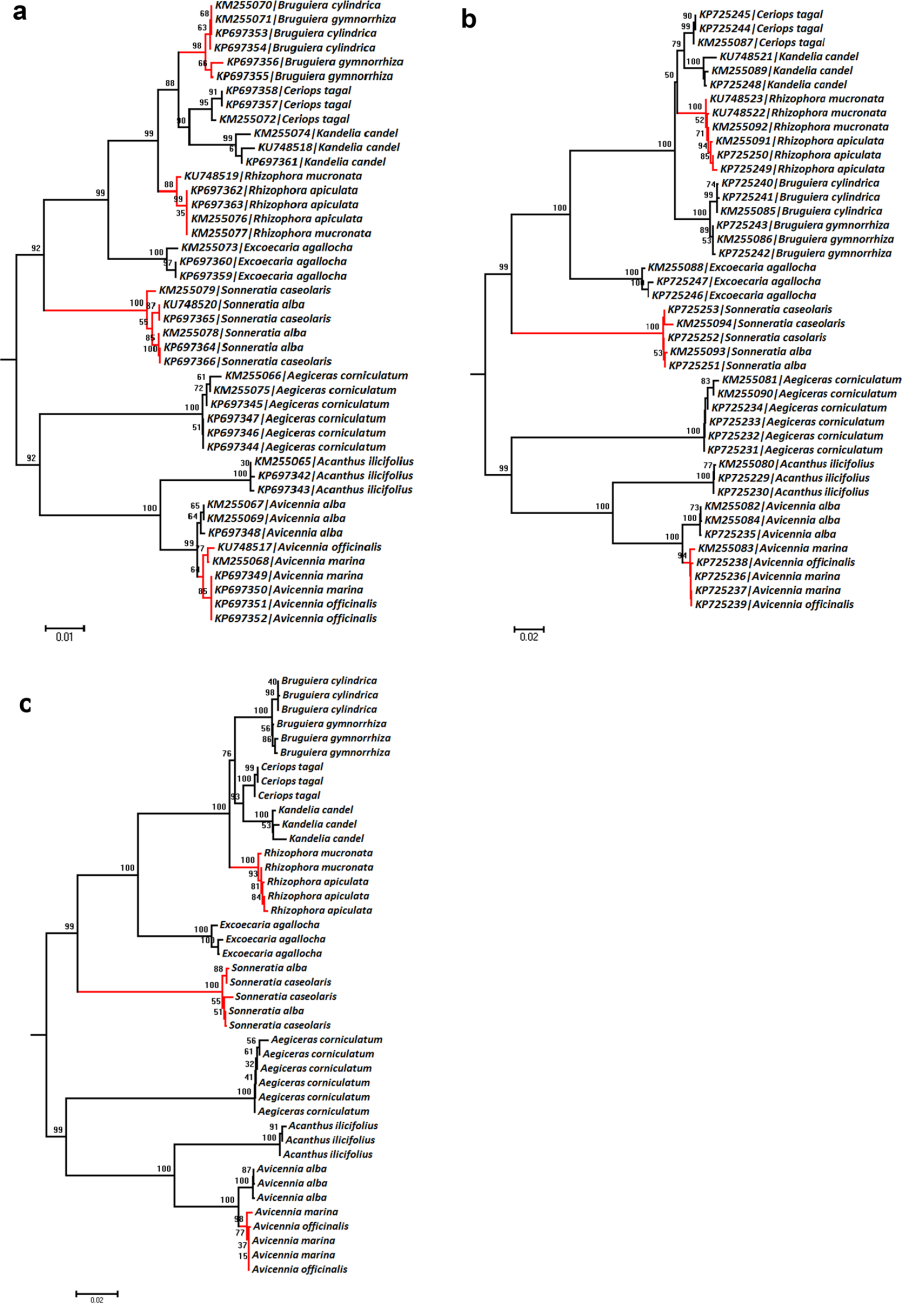
Neighbor joining tree (Kimura 2 Parameter distance using bootstrap value of 1000 replicates). **a** *rbcL,* **b** *matK,* and **c** *rbcL* + *matK* concatenated NJ (K2P) trees. Highlighted clades (*red color*) indicate unresolved or least differentiated mangroves sequences

The species were assigned to their taxa based on three methods, similarity based method using TaxonDNA, BLAST score based single linkage (BLASTClust) and tree based method (NJ). To assess the species assignment of single region and multi regions, we used the ‘Best Match’ (BM) and ‘Best Closest Match’ (BCM) criteria from TaxonDNA. For TaxonDNA analysis, we need to set threshold (T) below which 95 % of all intraspecific distances were found. All the results above the threshold (T) were treated as ‘incorrect’. Similarly, if all matches of the query sequence were below threshold (T), the barcode assignment was considered to be correct identification. The matches of the query sequence were equally good, but correspond to a mixture of species, then test was treated as ambiguous identification. For the single barcode region, *matK* had the highest rate of correct identification using BM (72.09 %) and BCM (39.53 %) than *rbcL* with (BM 47.72 %), BCM (31.81 %) (Table 3). The concatenated regions (*rbcL* + *matK*) demonstrated to resolve species at the level of 66.6 % using BM and BCM criteria (Table 3). The species specific clustering using match and mismatch criteria was evaluated in TaxonDNA and BLASTClust, where sequences with highest similarity and identity were considered as successfully identified. Those species with an identical barcode sequence to an individual of other species were considered as ambiguous, and sequences matching with different species names were treated as failure identifications. Species having single sample and unique sequence were considered as potentially distinguishable. The BLASTClust analysis revealed slightly different results than that of TaxonDNA, where the rate of species resolution and cluster formation was low as that of TaxonDNA (Table 4). Species with multiple individuals forming a monophyletic clade in NJ trees with a bootstrap value above 60 % were considered as successful identifications (Kress et al. 2010). The *matK* and *rbcL* + *matK* discriminated mangrove species in NJ model test method, while *rbcL* alone failed to identify those species (Fig. 2a–c). Further analysis revealed similar rates of species resolution using both methods for *matK* as well as *rbcL* (Table 5). *Rhizophora*, *Sonneratia* and *Avicennia* genera were failed to discriminate their species using plastid markers *rbcL, matK* and *rbcL* + *matK*.

**Table 3:** Identification success rates using TaxonDNA (Species Identifier) program under ‘Best Match’ and ‘Best Closest Match’ methods

Barcodes	No. of Sequences	Best Match (%)			Best closest match (%)				T (%)	No. of clusters	Match/mismatch
		Correct	Ambiguous	Incorrect	Correct	Ambiguous	Incorrect	No match			
*rbcL*	44	47.72	36.36	15.9	31.81	27.27	11.36	13	0	23	6/8
*matK*	43	72.09	25.58	2.32	39.53	13.95	2.32	44.18	0.11	24	10/4
*rbcL* + *matK*	42	66.66	16.66	16.66	66.66	16.66	16.66	0	0.2	21	8/6

TaxonDNA is an alignment-based method based on sequence distance matrices. Percentage of correct/incorrect/ambiguous assignment of a taxon is compared using molecular operating taxonomic unit (MOTU). The species specific clustering using match and mismatch criteria

*T* Threshold

**Table 4:** Identifications of all mangrove samples based on BLASTClust result

Barcode	No. of sequences	Average length of sequences	Number of species	Number of clusters	Match/mismatch
*rbcL*	44	586	14	6	3/11
*matK*	43	818	14	8	3/11
*rbcL* + *matK*	42	1404	14	15	4/10

BLASTClust is a method based on blast similarity scores of unaligned sequences

**Table 5:** Identification achieved by phylogenetic analysis using Neighbor Joining (NJ) and various methods, obtained from models test

Barcodes	Match/mismatch (NJ method)	Match/mismatch (Model test method)
*rbcL*	6/(8)	6/8 (K2 + G)
*matK*	8/(6)	8/6 (GTR + I)
*rbcL* + *matK*	8/(6)	8/6 (T92 + I)

For each, Bootstrap replicates = 1000

*K2* + *G* Kimura 2 + Gamma distribution, *GTR* + *I* Generalised time reversible + proportion of invariable sites (I), *T92* + *I* Tamura 1992 Model + proportion of invariable sites (I)

## Discussion

To the best of our knowledge, current study is the first attempt of performing DNA barcoding based assessment of mangroves from Goa using plastid core markers *rbcL* and *matK*. Some countable reports based on molecular taxonomy and phylogeny of Indian mangroves are available using nuclear, mitochondrial and plastid markers (ITS, *rbcL*, RFLP, RAPD, PCR-RAPD and AFLP) (Parani et al. 1997a, b; Lakshmi et al. 1997, 2000; Setoguchi et al. 1999; Schwarzbach and Ricklefs 2000). Besides this there are many reports of mangroves identification based on morphological characters (Untawale 1985; Tomlinson 1986; Untawale and Jagtap 1992). Present study revealed discrimination of mangroves based on DNA barcoding at species level excluding some taxa (*Rhizophora*, *Sonneratia* and *Avicennia*). Highest rate of PCR amplification and sequencing was observed in *rbcL* (97.7 %), while amplification as well as sequencing rate of *matK* was 95.5 %. Similarly, highest success rate of identification was observed with *matK* (80.5 %) in local temperate flora of Canada and in combination *rbcL* + *matK* identified 93 % flora (Burgess et al. 2011). Species identification success rate using *rbcL* seems to be higher, whereas *rbcL* recovery ranged from 90 to 100 % (Little and Stevenson 2007; Ross et al. 2008; CBOL Plant Working Group 2009). *matK* showed difficulties in PCR amplification and sequencing. Fazekas et al. (2008) showed that *matK* markers provide possibility of 88 % sequencing success, with the use of 10 primer pair combinations. Similarly, a lower amplification and sequencing success of *matK* has been reported in several other studies and amplification ranges from 42 to 70 % (Ford et al. 2009; Gonzalez et al. 2009; Kress et al. 2010; Hollingsworth et al. 2011). In contrast, CBOL reported that single pair of *matK* primer was successfully amplified and sequenced 84 % angiosperm species (CBOL Plant Working Group, 2009). We faced many hindrances in amplification and sequencing of *Rhizophora* genera species *R. apiculata* using universal *matK* primers. *R. apiculata* was amplified and sequenced using universal *rbcL* marker but for *matK* amplificaiton, we designed a reverse primer. The possible explanation for the trouble could be due to secondary metabolite might hindered amplification of target genes or failure of primers to amplify genes.

Initially, species identification was performed by NCBI BLAST using *rbcL* and *matK* sequence data, the BLAST could yield accurate identifications results (Hollingsworth et al. 2009; Kress et al. 2010; Kuzmina et al. 2012). On a similar note BLAST was performed revealing its least efficacy in species identification. It has been used for verification purpose in recent years and comparisons based on test datasets (Ford et al. 2009). Parmentier et al. (2013) reported that species assignment using BLAST method was reliable for genus identification of African rainforest tree (95–100 % success), but less for species identification (71–88 %). Sometimes it gave erroneous identifications, most often due to the limited number of available reference sequences. In the present study, BLAST result with default parameter, for *rbcL* successfully identified genera (100 %) and species identification rate was 64.28 % for 14 mangroves species. *matK* was able to identify genera (100 %) and species identification up to 85.71 % successfully. The possible reason for this erroneous assignment in some taxa at species level due to availability of limited sequences in the BOLD or GenBank database (Parmentier et al. 2013). Our result underscored the importance of BLAST method to assigned correct mangroves genera identification (with *rbcL* and *matK*). Both *Sonneratia alba* and *Avicennia marina* were incorrectly identified at species level using *rbcL* and *matK*. Some mangrove species viz. *R. apiculata*, *B. cylindrica* and *A. alba* were misidentified at species level using *rbcL.*

The genetic divergence analysis exhibited highest divergence in *Avicennia* species, while barcode gap and nearest neighbor analysis revealed low species resolution and barcode gap with nearest neighboring distance (<2 %), further confirming species overlap in *Avicennia* (*A. officinalis* (*rbcL*:0; *matK*: 0–1.71) and *A. marina* (*rbcL*: 0–0.34; *matK*: 0), *Bruguiera* (*B. gymnorrhiza* (*rbcL*: 0; *matK*: 0.61) and *B. cylindrica* (*rbcL*: 0–1.71; *matK*: 0.61), *Rhizophora* (*R. mucronata* (*rbcL*: 0; *matK*: 0.14) and *R. apiculata* (*rbcL*: 0; *matK*: 0.14), *Sonneratia* (*S. caseolaris* (*rbcL*: 0; *matK*: 0) and *S. alba* (*rbcL*: 0; *matK*: 0). Low genetic distances between species was largely due to the presence of species-rich genera with low sequence variation for the plastid genome (Burgess et al. 2011).

The species identification and taxon assignment was evaluated using TaxonDNA and BLASTClust for *rbcL, matK* and *rbcL* + *matK*. Overall *matK* marker showed good performance at species and genus level (Tables 3, 4). In contrast to *matK*; *rbcL* alone showed poor performance at species level identification. Combined, *rbcL* + *matK* markers showed better performance at species and genus level identification (Tables 3, 4, 5). Accordingly, plant CBOL group (2009) reported only 72 % species level resolution using combined *rbcL* and *matK*. Similar result was observed after combined *rbcL* and *matK* at species level resolution (Chen et al. 2015). Lowest resolution was recorded in closely related groups of *Lysimachia* with combination of *rbcL* and *matK* universal markers (Zhang et al. 2012). However, the identification rates based on TaxonDNA and phylogenetic tree methods (Tables 3, 5) were significant with *matK* as compared to *rbcL.* Low resolution using DNA barcoding regions has been documented in many other plants such as the genus *Araucaria* (32 %), *Solidago* (17 %) and *Quercus* (0 %) (Little and Stevenson 2007; Leon-Romero et al. 2012). In TaxonDNA analysis, for *rbcL* threshold (T) was observed 0 %, similar result was recorded for *rbcL* in the Zingiberaceae family (Chen et al. 2015). However, threshold (T) for Indian Zingiberaceae family members were recorded as 0.20 % for *rbcL* and 0 % for *rpoB* and *accD* (Vinitha et al. 2014). In BLASTClust, the *rbcL* and *matK* regions showed similar identification rates, while concatenation of both these regions increased the efficiency of species resolution as well as cluster formation (Gonzalez et al. 2009; Blaalid et al. 2013). In case of closest taxa of mangroves viz. *Avicennia*, *Rhizophora* and *Sonneratia* species, there is a need to explore new DNA barcode markers, which may leads to species level resolution.

## Conclusions

DNA barcoding can be a very effective tool to identify mangroves. Here, we tested DNA barcodes of plant plastid DNA, *rbcL* and *matK* to resolve available mangrove species. For the single barcode region, *matK* had the highest rate of correct identification using BM and BCM than *rbcL*. When both regions were concatenated (*rbcL* + *matK*) their efficiency to resolve species was 66.6 % using BM and BCM criteria. In the present work, we lay the foundation towards DNA barcoding applications for mangroves plant genera viz. *Acanthus, Kandelia*, *Ceriops*, *Bruguiera*, *Aegiceras* and *Excoecaria. matK* is proposed to be a suitable candidate DNA barcode marker for mangrove species identification. Compiled mangroves barcoding result had some limitations, most of which are due to imperfect discrimination ability of the markers, natural hybridization and homoplasy. Further need to explore with additional markers which may improve mangrove species identification for practical conservation.

## Additional file

10.1186/s40064-016-3191-4 Photos of 14 mangroves species. **Table S1**. Morphological key features.

## Abbreviations

*RbcL*: ribulose bisphosphate carboxylase large subunit
*matK*: maturase K
