# Supplementary material for: Assessment of mangroves from Goa, west coast India using DNA barcode
Source: Springerplus. 2016 Sep 13;5(1):1554. doi: 10.1186/s40064-016-3191-4 (PMC5021661; doi:10.1186/s40064-016-3191-4)
Supplement: Supplementary file 1 — 10.1186/s40064-016-3191-4 Photos of 14 mangroves species. Table S1. Morphological key features. [file 40064_2016_3191_MOESM1_ESM.docx]

**
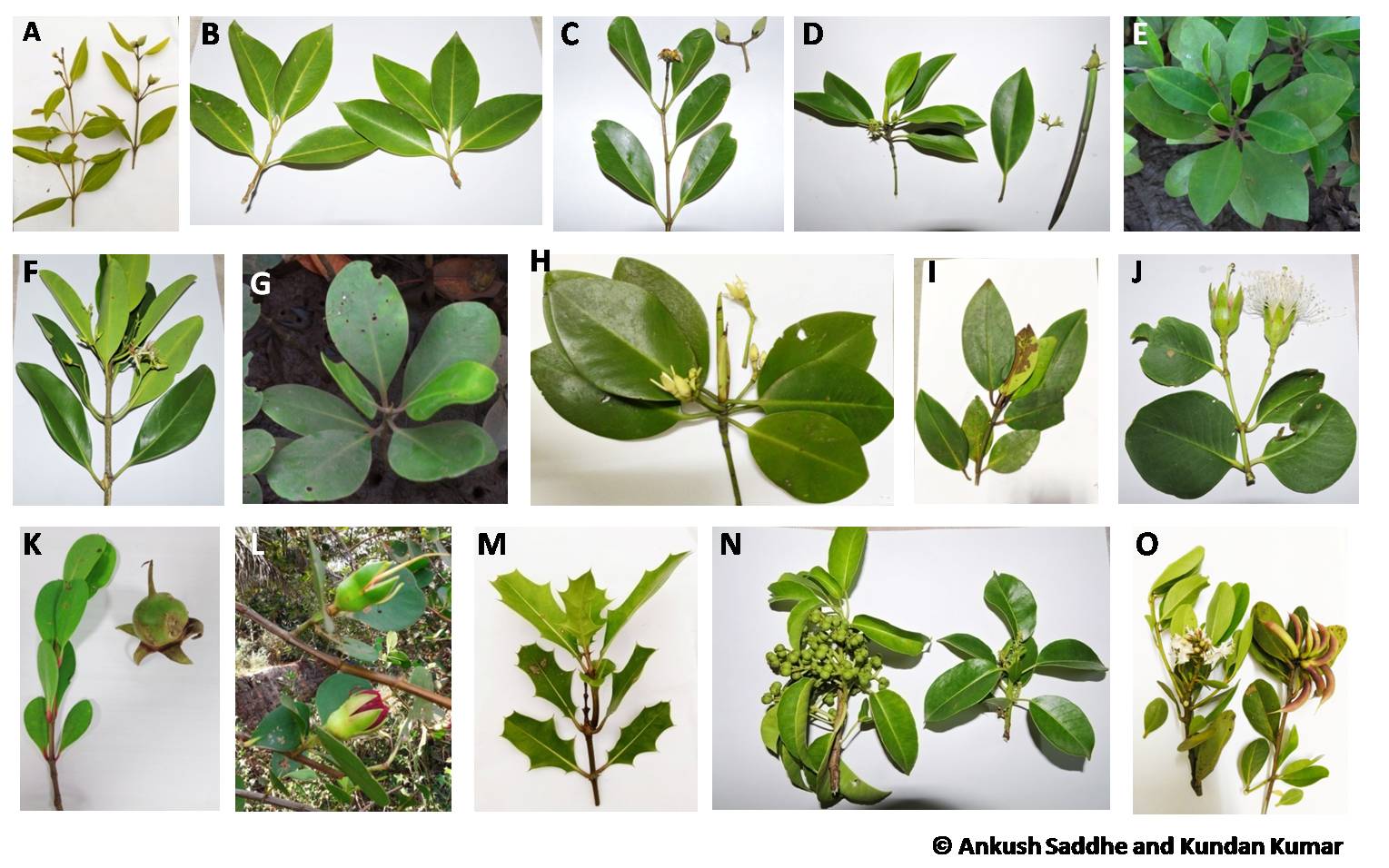
Figure S1**. Photos of 14 mangroves species used in our study (A) *Avicennia marina* (B) *A. alba* (C) *A. officinalis* (D) *Bruguiera cylindrica* (E) *B. gymnorrhiza* (F) *Kandelia candel* (G) *Ceriops tagal* (H) *Rhizophora mucronata* (I) *R. apiculata* (J) *Sonneratia alba* (K, L) *S. caseolaris (*M) *Acanthus ilicifolius* (N) *Excoecaria agallocha* and (O) *Aegiceras corniculatum.*

**Table S1**. **Morphological key features of 14 Mangroves species**. Morphological identification of mangroves species based on leaves, flowers and fruits. Key features in bold in bold indicates the differentiating characters used to distinguish in the study.

*References not cited in literature

Duke CN (1991) A Systematic Revision of the Mangrove Genus Avicennia (Avicenniaceae) in Australasia. Aust Syst Bot 4:299-324

Duke CN, Bunt SJ (1979) The Genus Rhizophora (Rhizophoraceae) in North-eastern Australia. Aust J Bot 27:657-78

Duke CN, Jackes RB (1987) A systematic revision of the mangrove genus Sonneratia (Sonneratiaceae) in Australasia. Blumea 32:277-302
